# Referred Sensations Elicited by Video-Mediated Mirroring of Hands

**DOI:** 10.1371/journal.pone.0050942

**Published:** 2012-12-18

**Authors:** Simon Hoermann, Elizabeth A. Franz, Holger Regenbrecht

**Affiliations:** 1 Department of Information Science, University of Otago, Dunedin, New Zealand; 2 Department of Psychology, University of Otago, Dunedin, New Zealand; Weill Cornell Medical College, United States of America

## Abstract

Humans readily perceive ownership of a limb even when it is artificially induced as in the case of using a mirror reflection. However, mirror reflections are very constrained perceptions which do not allow transformations and varied contexts as often occurs in real life. The extent to which perceived limb ownership occurs with video-mediated manipulations is not known, particularly given the perception would no longer be a precise copy (reflection) of a person’s own limb. The present study directly compared referred sensations of the limbs with the use of a mirror reflection to those obtained with a new video-mediated setup to assess perceived ownership. Manipulations that could not be performed with a standard mirror reflection, such as reversal of the spatial positions of the limbs, were also investigated to examine how far the perceived ownership effects could be pushed. Across a series of experiments, data on the quality, intensity and location of referred sensations were collected and analyzed together with measures of hand ownership and participants’ experience of the two setups. Results reveal that participants felt referred sensations in both the optical and the video-mediated setup, and that video-mediated manipulations of hand-position reversals produced equal to stronger effects of ownership compared with the mirror reflection. These findings open up new possibilities for scientific experimentation and therapy that are discussed in the paper.

## Introduction

Mirror visual feedback (MVF) as a possible therapy was first introduced in 1995 [1], and has since been used for the treatment of a variety of neurological disorders, including phantom limb pain [2], [3], stroke [4], pain related to spinal cord or nerve injuries [3], following wrist fracture [5], fibromyalgia [6], complex regional pain syndrome (CRPS) type 1 [7]–[11] and CRPS type 2 [12]. The main component of the MVF manipulation is a mirror vertically propped up in a position between two adjacently positioned boxes. Parts of the front of the boxes are removed to allow the patient to place one or both hands separately inside the boxes, and parts of the tops of the boxes are also removed to allow the patient to view one hand and its mirror image (reflection) simultaneously. Viewing the healthy fully functional hand and its mirror reflection creates the visual impression of bimanual movements. Recent applications using the MVF have also incorporated referred tactile sensations in a mirrored hand, an experimental possibility that could lead to critical understanding about how visual and sensory sensations combine in the brain, and ultimately, advances in therapies implementing such approaches. However, given that experimental manipulations are rather constrained with the use of the standard mirror box (such as subject biases and expectations), it is rather difficult to test hypotheses about the precise nature of the observed effects. The present study begins to examine such hypotheses with the use of a video-mediated setup. Before describing our own study, we will briefly cover some related research which led to our own questions.

Referred sensations, or sensations felt on sites on the skin which were not actually stimulated, have been studied in the context of mirror therapy since about 1996 [2]. However, initial studies were unable to induce intra-manual referred tactile sensations in healthy participants and concluded that the effects of referred sensations are unique to phantom limbs. Similarly Sathian [13] did not find contralateral referred sensations in normal subjects or in hemiparetic patients without sensory loss affecting the hand. In arm amputees however, Ramachandran & Hirstein [14] were able to elicit RS in 4 out of the 10 participants. In another study by the same first-author it was reported that RS was evoked from the face to an amputated limb [15].

Several other studies have also demonstrated the presence of RS, for example in a poststroke patient [16], in six patients with hands anesthetized by stroke or neurosurgery [13] and in two subjects with spinal-cord injury [17]. Studies on patients with CRPS 1 have also shown evidence of referred sensations. In particular, McCabe et al. [18] reported tactile RS in 5 out of 16 subjects, with closed eyes, but not when participants had direct vision of the stimulated limb. Acerra & Moseley [19] conducted a study with patients suffering from CRPS type 1 and used a mirror to superimpose the reflected stimulated image of the healthy limb over the affected one. They found that if areas of the unaffected limb were stimulated, patients could feel pain in the affected side if the corresponding area was affected by allodynia, and patients felt “pins and needles” or tingling if that side was affected by paresthesia. However, that study did not report that patients experienced any RS on unaffected parts of their limb(s). In contrast Krämer, Seddigh, Moseley et al. [20] were not able to evoke RS in (non-CRPS) chronic neuropathic pain patients with brush-evoked pain (allodynia), using a similar method.

Of special interest to our study is the work by Takasugi et al. [21] in which RS was assessed in two experiments with healthy participants. In the first experiment, 21 participants were queried about RS in their own masked hand behind a mirror, while observing the reflected image of their stimulated other hand (control condition) and then the reflected image of the stimulation of an assistant’s hand (experiment condition). In the second experiment, with 16 participants, the hand of the assistant was replaced with a rubber hand (experiment condition) while the control condition was the same. The researchers were able to elicit RS in all conditions with the experimental condition significantly stronger than the control condition in both experiments. They also reported ownership feelings associated with the visual appearance of the hand in the mirror image in the experimental condition in all but one participant in the first experiment, and in all participants in the second experiment.

Research of perceived ownership of virtual or artificial limbs has also been the focus of various studies. Among others, Botvinick & Cohen [22] analyzed the Rubber hand illusion (RHI), i.e. the perceived ownership of an artificial hand which was simultaneously stimulated with a participant’s occluded hand placed next to it. This study provided the basis for other studies. Pescatore et al. [23] and IJsselsteijn, de Kort, & Haans [24] analyzed ownership and the effects of the RHI in a real and a virtual reality setup. Pescatore et al. [23] used the questionnaire from Botvinick & Cohen [22] whereas IJsselsteijn et al. [24] extended it for their study. Durgin et al. [25] used their own questionnaire for participants in three different setups where the stimulation of the rubber hand was either observed directly or mediated through a camera and a projector, or the projected rubber hand was stimulated in front of the participant. In addition, different orientations of the rubber hand as well as the use of red laser light instead of the brush were used. The study found that overall two thirds of the participants reported feeling somatic sensations from the laser light. But no direct comparison of standard mirror reflections and video-mediated manipulations have been conducted across a series of RS situations such as those demonstrated in Takasugi et al. [21]. Understanding whether referred sensations are possible across tasks which are impossible to test using a standard mirror box (i.e., reversal of the positions of the limbs/hands), is critical in the further advance of therapies for movement rehabilitation and will further inform studies into the neural processes underlying such effects.

We first addressed the question of whether perceived ownership and the intensity and quantity of referred sensations might be enhanced with the use of a video-mediated system (ART) which we have purposely-designed to overcome some of the constraints of a standard optical mirror box (OMB). Specifically, our ART setup enables us to mirror both hands using computer transformations of their images, something not possible using the OMB. This allows us, for example, to eliminate the possible effects of subject bias in that participants clearly know which hand is being mirrored when they engage in a standard experiment with an OMB. Furthermore, with the OMB the precise type of mirroring (a form of symmetrical bimanual reflection) is virtually the only one possible, and the participant is well aware of this. We can again overcome this with our use of the ART, thereby removing expectations associated with the participant’s knowledge and experience with standard mirror effects. This led us to wonder whether, with removal of such constraints inherent with use of the OMB, we might be able to find even stronger effects of image transformations (with our ART setup) suggesting that we can further ‘fool the brain’ and thereby open up additional avenues for therapeutic manipulations as well. Moreover, such findings would open the door to much-needed studies that would dissociate influences of hand dominance and differential distribution of attention to the two hands, on efficacy of unilateral therapies for rehabilitation of disorders and traumatic injury. In the present study, we limit our manipulations to those using referred sensation (RS), with the initial hypothesis that effects with the flexible adaptations and transformations with the video-mediated setup (to be explained) will result in enhanced effects of RS in comparison with the OMB.

## Materials and Methods

### Ethics Statement

The experiment was approved by the University of Otago ethics committee. Informed written consent to participate in the study was obtained from all participants.

### Participants

Twenty-one healthy volunteers (7 female, 14 male; mean age ± SD, 27.81±4.82) participated in the experiment. Handedness (L.Q.) was calculated with the Edinburgh Handedness Inventory [26] and 4 participants were classified left-handed (mean L.Q. ± SD, −94±12) and 17 as right handed (mean L.Q. ± SD, +98±6). All participants had, as required by our inclusion and exclusion criteria, normal or corrected to normal vision and reported to have no disability in their hand, arm, shoulder, neck, back or other areas that could have affected their performance.

### Apparatus

**Figure 1:**
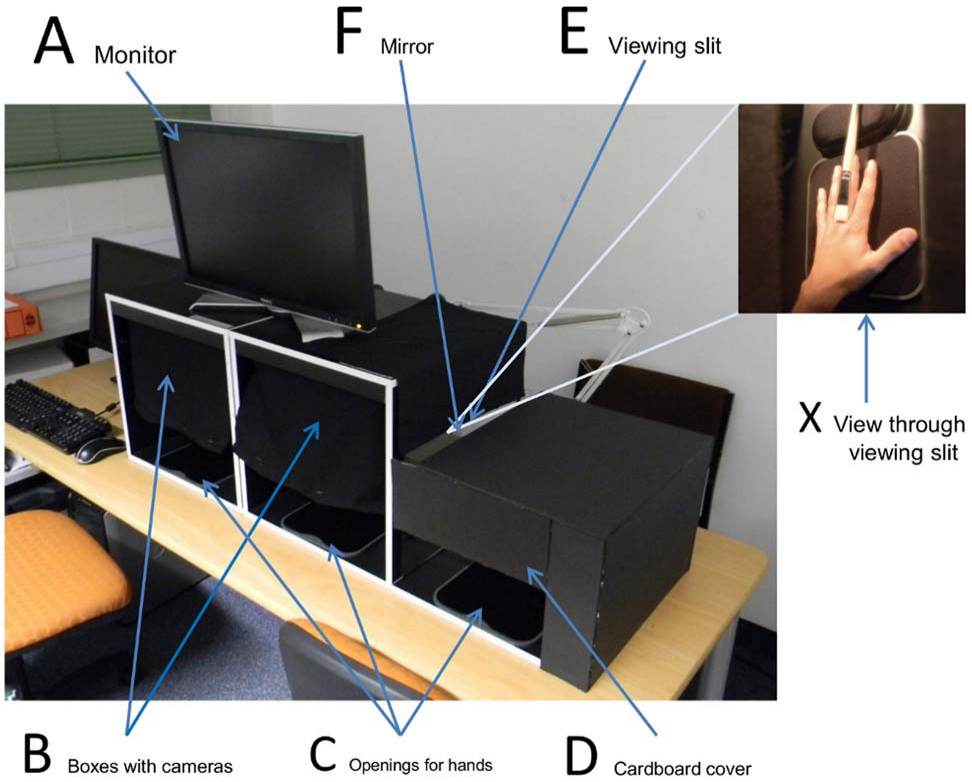
Experimental setup. For the conditions ART and advART a monitor (A) is used which displays the reflected hand(s) to the participants. Left and right boxes (B) block visibility of the hands for the participants and capture the hand movement with cameras. The box on the right hand side is also used to hide the left hand (without capturing) for the OMB condition, in which the participants place the hand into the opening (C) where a cardboard cover (D) blocks the view to the hand. A viewing slit (E) allows participants a clear view of the reflection of a hand without seeing the actual hand. Brushing stimulation (X) takes place under the cardboard cover (D).

The apparatus consists of a setup (Figure 1) that combines both the video-mediated system (left part) as well as the optical mirror box (right part) next to each other in a single unit. The optical mirror box (OMB) part was constructed based on the description from [21]. It consists of a cardboard box allowing only the view of the reflected right hand, through a square viewing slit on the top, in a perpendicular mirror in the centre and occluding the direct view of the right hand, with the purpose to create the impression that the hand seen in the mirror is the left one. To increase comfort and to allow uniform positioning of the hands of all participants in the indicated area, black wrist supports, normally used as computer mouse pads, were placed inside the boxes at a central position (Figure 1x).

The OMB was constructed out of black cardboard. The size of the box is 37 cm wide, 26 cm high and 40 cm deep and has a 5×40 cm viewing slit on top. The wooden planks underneath both setups are 1 cm thick. The opening in front of the box is 29×11 cm, which is large enough to comfortably place hands of any size inside the box but also just high enough to obstruct the participant’s direct view of the hand, to avoid direct visual feedback which was reported to diminish or prevent RS in CRPS1 patients [8]. The visible part of the mirror on the left side of the OMB is attached to the outer side of the other setup and measures 35×25 cm.

**Figure 2:**
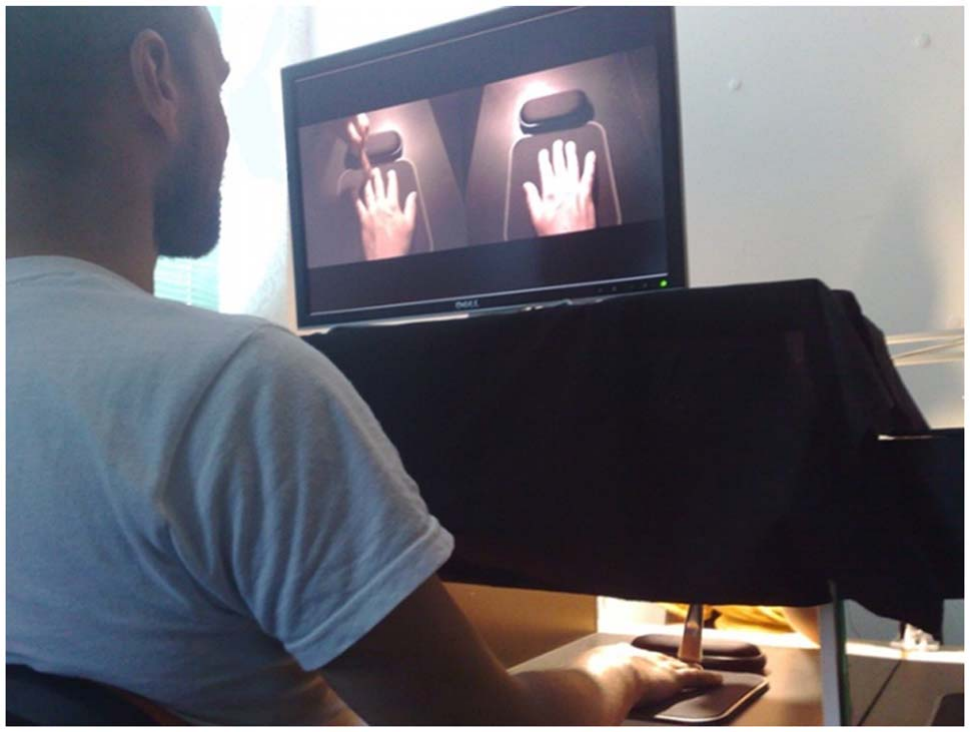
The video-mediated system as used for the advART condition. In this (advART) as well as in the other two condition the participant sits in front of the system and observes the mirrored images of his hand(s) either on the screen or in the optical mirror while an assistant is brushing the participant’s right hand. In the advART condition, shown in this picture, the participant sees the mirror image of his actually brushed right hand on the left side of the screen while the mirror image of his left hand is shown on the right side of the screen.

The computer video-mediated setup, i.e. the Augmented Reflection Technology (ART) system, consists of two black wooden boxes of 40×39×40 cm size, which is approximately the same length and width as the OMB. The hole for the hands in front of the boxes is 37×11 cm and a curtain instead of cardboard was used to limit the participant’s view. A webcam with a wide angle lens (Philips SPC1000NC) was placed in each box and recorded the hand(s) with a resolution of 640×480 at 30 frames per second. On top of the boxes a 22 inch widescreen monitor (Dell UltraSharp 2208WFP) running at the native 1680 x 1050 resolution @ 60 Hz was used as the display (Figure 2). A detailed description of the system is provided in [27].

A short handled hog hair flat style brush of the size 16 (used normally for poster, acrylics, oils and tempera paint) was used as a brushing device in the experiments.

### Experimental Settings

**Figure 3:**
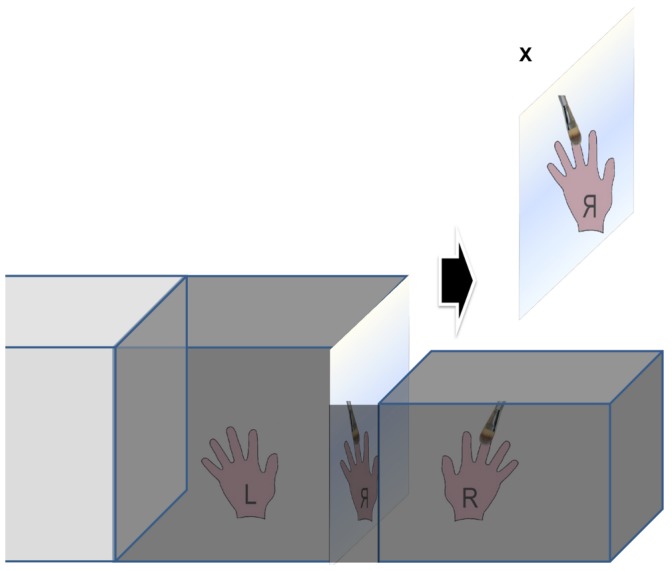
OMB condition. In the OMB condition the optical reflection (mirroring) of the right hand, which is stimulated with brushing strokes, is observed through the viewing slit. The left hand is hidden in the neighboring box (behind a curtain out of the view from the participant).

Three experimental conditions were used: The optical mirror box condition (OMB), the video-mediated Augmented Reflection Technology condition (ART) and the video-mediated advanced Augmented Reflection Technology condition (advART). In the OMB condition shown in Figure 3 participants placed their hands in the outer right cardboard box (right hand) and the wooden box in the middle (left hand). The left hand in the wooden box was completely hidden from the participant’s view. Participants could only observe the image of their right hand in the mirror (Figure 3x) through the viewing slit in between the two boxes. The right hand was brushed by an assistant sitting behind the system.

**Figure 4:**
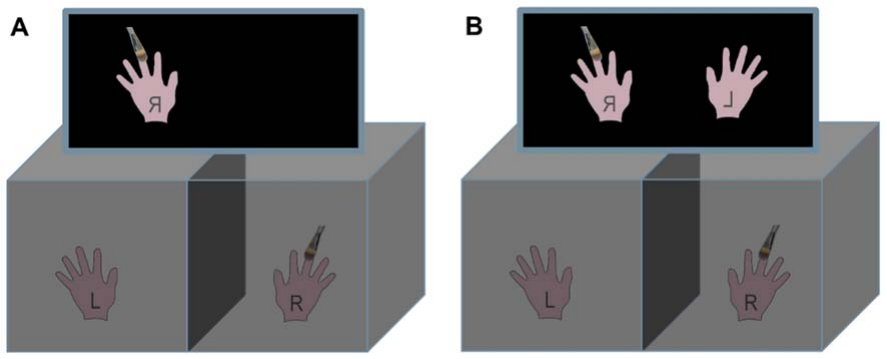
ART (A) and advART (B) conditions. In both the ART (A) and the advART (B) conditions the participant’s right hand is stimulated with a brush. This stimulation is not directly visible to the participant. A mirrored image of the right hand is presented on the left side of the screen. In the advART (B) in addition to the mirrored right hand on the left side also the mirrored left hand is shown on the screen.

In the ART and advART condition participants placed their hands inside the two black wooden boxes. Both hands were hidden from the participant’s direct view. Participants were instructed to observe only the hand(s) on the screen. In the ART condition participants were shown only the mirrored and brushed right hand on the left side of screen (Figure 4a) whereas in the advART condition also the mirror image of the left hand was shown on the right side of the screen (Figure 4b). Only the right hand was stimulated with a brush.

### Measures

Assessments of referred sensations (RS) were measured with verbal questions and a printed questionnaire. To measure RS, the assessment was focused on:

1. the experience of sensation on the masked hand with the question “Can you tell me what you felt on your left hand?”
2. the intensity of sensation (0 to 10 points scale), and
3. the location of the sensation if the participant previously reported RS.

A separate questionnaire was handed to participants at the end of the last condition that included questions about:

1. Socio-demographic characteristics, such as age and gender,
2. Handedness (using the Edinburgh Handedness Inventory [26]) and self-reported handedness (current and past)
3. Use of computer games,
4. Hand ownership in the different setups with the question “I had the feeling that the hands [in the Mirror/on the Screen] are my hands” on a 7 point scale
5. Prior knowledge of this or similar systems,
6. Ratings of comfort and motivation (on a 7 point scale) with questions such as “How comfortable did you find the [Optical Mirror/Computer Monitor] setup” and “Could you imagine to motivate yourself to use the [Optical Mirror/Computer Monitor] setup every day for 10 minutes?”,
7. Direct comparison between the two systems with the question: “What equipment would you prefer to use regularly?”.

### Experiment Procedure

All participants were asked to read and sign an Information sheet and consent form approved by University of Otago ethics committee. They were then explained the specific procedures, although were not told details about the different setups other than the obvious ones they could visually observe.

The experiment had three conditions; the OMB condition which was a replication of the control condition in Takasugi et al. [21]; the ART condition which simulated the effect of a mirror on the screen; and the advanced ART (advART) condition which made use of one of the new possibilities with ART, i.e. the cross-mirroring effect, which in addition to the mirrored and stimulated right hand seen on the left side of the screen, also displayed the mirrored left hand and not stimulated on the right side of the screen (Figure 2). This type of manipulation is impossible with the OMB and enables us to assess whether people really can identify which hand is which without other contextual cues.

All participants took part in all three conditions with the order of the first and second condition randomized between the OMB and the ART condition, and the third always being the advART (cross-mirroring) condition.

Participants were asked to place their hands into the boxes after removing any jewellery. The leftmost and the middle boxes were used for the ART and advART condition, whereas the middle and the right boxes were used for the OMB condition (Figure 1). The participants were instructed to adjust the seat to a comfortable height and look into the mirror or on the screen, depending on condition. They were asked to not move their hands and fingers during the stimulation of their fingers with the painting brush.

The brushing was a light touch at the pace of 1 Hz performed by a highly-trained assistant sitting behind the boxes, using his left hand. Each finger of the right hand was stimulated 25 times in each condition. The order of the stimulation of the fingers was randomized for each participant and was kept the same across the conditions within a participant. The stroke of the brush started at the knuckle and ended at the fingernail. The assistant practiced this procedure extensively before applying it to participants.

Participants were asked to describe after each condition, the experienced sensation they felt on the masked (and therefore unseen) left hand. Participants were asked to report on the intensity of the perceived sensation on a scale of 0 (no sensation) to 10 (normal touch, corresponding to the intensity of actual brushing).

Before the main part of the experiment, a warm-up session was conducted using brush strokes and no visual feedback to ascertain that participants understood what they were being instructed to do (i.e., report referred sensations on the left hand and not what they felt on the right hand). After such instructions and procedures were clear, the main experiment began.

As mentioned previously, the main part of the experiment consisted of the three conditions, OMB, ART, and advART. In the OMB condition participants had to observe their mirrored and stimulated right hand through a looking window (viewing slit). This slit was used to obstruct participants’ views of their right hand and to make sure that only the hand in the mirror could be seen. Takasugi et al. [21] used the same approach in their experiment. The ART condition replicated the mirror condition but instead of the mirror, participants were asked to observe the mirrored image of their right hand displayed on the left side of the computer-screen (Figure 4a). In the advART condition, in addition to the mirrored right hand on the screen on the left side, the mirrored left hand was also simultaneously displayed on the right side of the screen (Figure 4b). The order of the ART and OMB conditions was quasi-randomized with 12 participants starting with OMB and 9 with ART.

After the end of the last condition participants were asked to fill out the questionnaire. A short debriefing, where participants were informed that their hand was mirrored and asked about their general experience, concluded the experiment. Participants received a large chocolate bar for agreeing to participate at the experiment.

### Statistical Analysis

The data were analysed with Microsoft Excel 2010 SP1. Inferential statistics were calculated using t-Tests (paired sample) from the Analysis ToolPak Add-in. Scores across all fingers were taken for the analysis of quantity and intensity of RS. The data from other experiments were either taken directly from the text in the papers, or in case of the standard error, were calculated from given variance or standard deviation. In cases where no precise values were mentioned, approximations of values are calculated from the charts in those papers.

## Results

The data analysis showed no significant difference between the optical mirror box (OMB) and the video-mediated ART in reported quantity and intensity of elicited referred sensations, thereby providing proof of concept in using our new ART. Furthermore, results from the questionnaire on perceived ownership of hands, comfort of use, motivation to use, also did not show any significant differences between the video-mediated ART system and the OMB, although the ratings for ART were on average higher than for OMB. In addition, preference of use and perceived therapy potential was rated by most participants as higher for ART. All participants reported that they were fully aware of observing a mirror image (or video-mediated image in the case of ART) while their fingers were being brushed.

### Perceived Ownership

**Figure 5:**
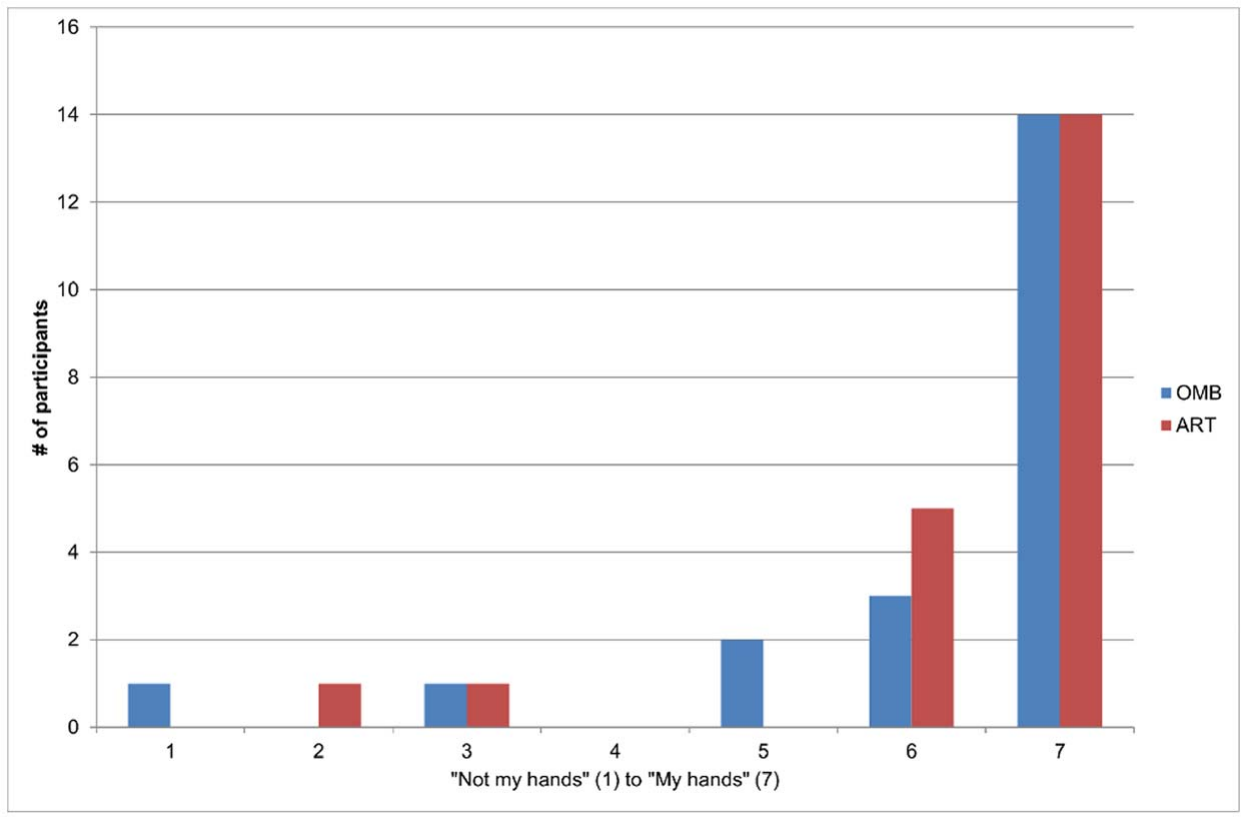
Perceived ownership of displayed limbs on the screen (ART) and on the mirror (OMB). In this histogram the perceived ownership rating of the displayed hand is shown for the two setups. The majority of participants perceived the hand shown in both setups to be their hand and rated it a 7 on a 7 point scale.

Most participants reported strong sensation (≥6), on a 7 point scale, that the image of the hand(s) they observed was one of their own hand(s). Specifically, 19 subjects (90.5%) in the ART condition and 17 subjects (81%) in the OMB condition reported strong sensation (Figure 5). Only one person (4.8%) rated that the hand(s) were not their own hands in both conditions, whereas one person (4.8%) had a slight negative sensation of ownership in both conditions and two people (9.5%) had only a slight sense of ownership in the OMB condition. Although the average perceived ownership for the video-mediated ART system was higher (ART: M = 6.33, SD = 1.35, OMB: M = 6.19, SD = 1.57) the difference was not statistically significant.

### Experience

Perceived comfort of use was rated positive by most participants. Seventeen (80.95%) in OMB and eighteen (85.71%) in ART participants gave positive ratings on a seven point scale from “Very uncomfortable” (−3) to “Very comfortable” (3). Three participants (14.29%) perceived the use of both setups as uncomfortable (<0 rating) and one person gave a neutral rating for the OMB setup (0). There was no significant difference in the rated comfort levels between the setups.

Thirteen participants (61.9%) could imagine motivating themselves to use either setup. Only 2 participants (9.52%) in the ART and 3 participants (14.29%) in the OMB setup gave negative ratings. Neutral ratings were given by 4 participants (19.05%) for the OMB and by 2 participants (9.52%) for the ART. Although the ratings for the video-mediated ART (M = 1.67, SD = 1.43) were on average higher than for the OMB (M = 1.24, SD = 1.64) the difference was not statistically significant.

Seventeen participants (80.95%) preferred the ART setup over the OMB setup. The average rating, on a 7 point scale from −3 (strongest preference for ART) to +3 (strongest preference for OMB), was −1.571. Two people (9.52%) preferred the OMB and 2 people (9.52%) did not prefer either setup. This result shows a highly significant (p<.001) preference of the ART compared to the neutral midpoint (0) on our rating scale.

**Figure 6:**
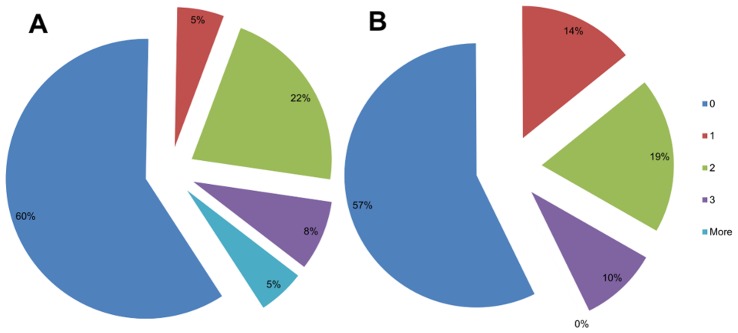
Comparison of reported referred sensation (RS). The classification of intensities of RS sensation in the study by Takasugi et al. [21] (A) and for our own study (B) using a similar OMB setup. The figure shows that the number of people who experienced RS is similar in both studies (40% and 43% respectively).

In the two experiments by Takasugi et al. [21] a total of 15 out of 37 (40.5%) participants experienced RS in the optical mirror setup (5/21 in the first, and 10/16 in second experiment) (Figure 6a). In the present study 9 out of 21 participants (42.9%) experienced referred sensations in the OMB (Figure 6b) which suggests that around 40% of people experience RS and showed that our data are in line with the results of Takasugi et al. [21].

**Figure 7:**
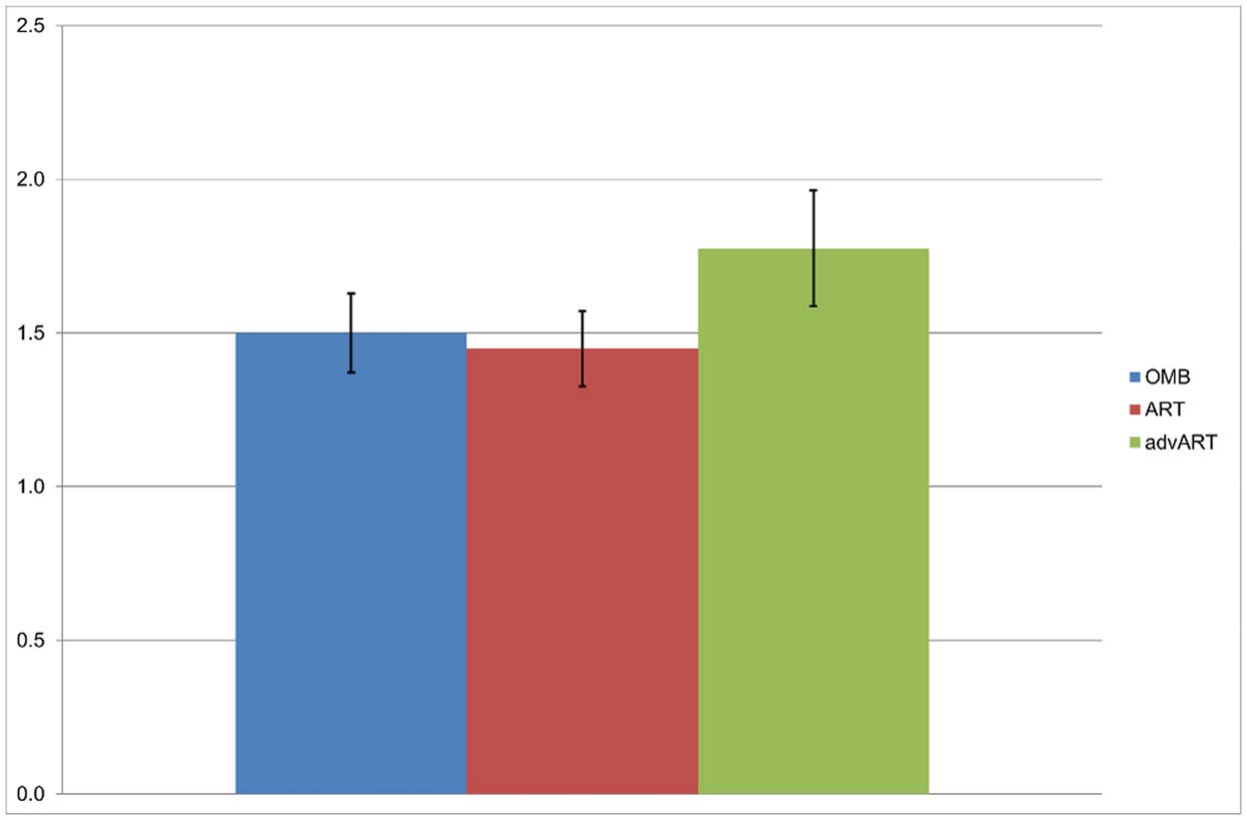
Comparison of RS intensities (Mean and Std-Error) between the three conditions. The figure shows the average intensities of RS felt by participants in the OMB (M = 1.5), ART (M = 1.45) and advART (M = 1.76) condition. (participants who did not report on experiencing RS in any condition were excluded).

In Figure 7 the averages of the RS intensities among the different setups are shown for all participants who reported to have felt RS at least once in one system (i.e. 10 participants). It can be seen that the average for the advART is higher than the average for the OMB and the ART conditions.

The statistical analysis comparing the reported RS between the three conditions across all data-points showed no significant difference between the OMB and ART. However, the comparison of advART with OMB was significant (t = 2.50, p = .01) as was its comparison with the regular ART (t = 2.77, p<.01), revealing significantly higher intensity ratings for the advART over the other conditions.

Almost all reported RS could be precisely localized by participants (97.96%, i.e. 96 out of 98 reported sensations) and were experienced at the same finger in the opposite hand as the stimulated finger (86.73%, i.e. 85 out of 98). This is in line with [2] who reported that in three of the four arm amputees who reported feeling RS, the touch of a finger was perceived exactly at the same position on the opposite phantom limb.

## Discussion

In this study we found that the results for RS and ownership of hands in the optical mirror box (OMB) are similar to those found with the video-mediated ART system. The values for perceived ownership are in alignment with the results from Takasugi et al. [21], where 20 (95.2%) subjects reported that the mirror image of the assistant’s hand evoked an ownership feeling and 16 (100%) of the subjects of the second experiment described in the same paper, reported that the mirror image of the rubber hand evoked an ownership feeling. Botvinick and Cohen [22] also reported that most participants (16/20, 80%) who observed a rubber hand synchronously brushed with their own occluded hand felt that the rubber hand was their own hand (ratings ≥5).

**Figure 8:**
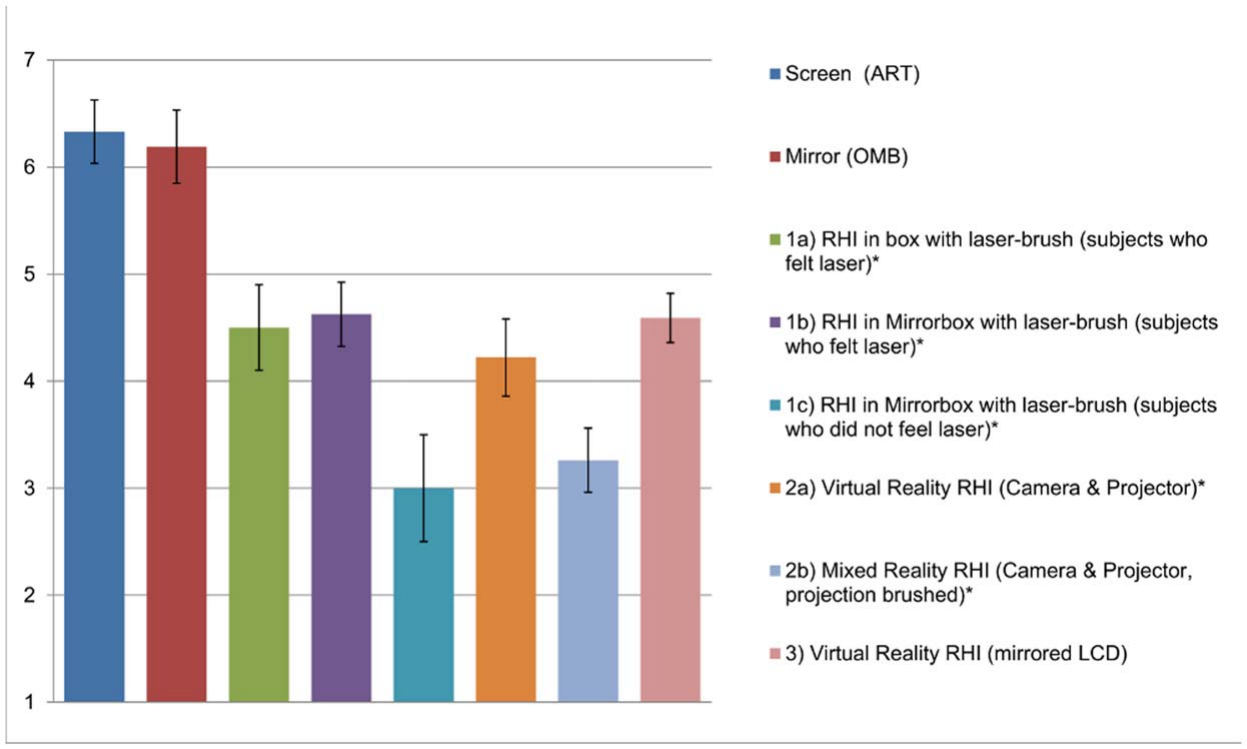
Ownership comparison with other experiments. The two leftmost columns show the ownership ratings obtained with our ART and OMB setups. All other columns show ratings found in the related literature: 1a) RHI in box with laser-brush (subjects who felt laser)*; 1b) RHI in optical mirror box with laser-brush (subjects who felt laser)*; 1c) RHI in optical mirror box with laser-brush (subjects who did not feel laser)*; (as in [25]) 2a) Virtual Reality RHI (Camera & Projector)*; 2b) Mixed Reality RHI (Camera & Projector, projection brushed)*; (as in [24] 3) Virtual Reality RHI (mirrored LCD) (as in [23]). *approximated values.

Compared to other studies in literature which used other setups to visually mediate tactile stimuli, the ratings of the felt ownership in our setups (i.e. ART (Screen) and OMB (Mirror)) exceeded the values reported for experiments using a similar OMB setup performed with rubber hands as shown in Figure 8. The bars 1a, 1b and 1c are results taken from Durgin et al. [25], 2a and 2b are from IJsselsteijn et al., [24] and 3 is from Pescatore et al. [23]. IJsselsteijn et al. [24], Durgin et al. [25] and Pescatore et al. [23] asked their participants to rate the question “I felt as if the rubber hand were my hand”. Note that the value for the original RHI experiment [22] on ownership (question number 3) is 6.3 with a range from 5 to 7.

These findings speak to the efficacy of our new ART setup as being a potentially valuable tool to induce ownership perception, which is perhaps a prerequisite of obtaining positive therapeutic outcomes. Given that our ART setups require immediate projection of camera-recorded information of the hands, rather than a mirror image reflection, these findings further speak to the ability of our setup to produce veridical images in a realistic timecourse to provide effects that are similar, and in most cases even stronger, than the standard OMB. Importantly, we have demonstrated referred sensations in perceptions of limbs that are actually in a different position from where they normally would be, a novel finding in the context of existing research which lends strong support for the view that perception (and not motor sensation) dominates in the brain’s ability to perceive the body, at least when such perception is visual.

The higher intensities of RS in the advART setup are unprecedented, as there has been no previous setup that can dissociate actual hand from visually-presented hand, nor has the manipulation of presenting one hand alone (our ART condition) versus presenting one hand by the other (our advART condition) been performed previous to this study. We clearly show that RS further depends on the appearance of the virtual other hand (i.e. both hands visually present compared to only one hand visually present) which is a novel and important (as well as interesting) finding in the present study. Note also, that this could not be accomplished using a standard mirror box setup; thus, we provide initial data showing a manipulation and finding not possible with the OMB and not previously shown. Theoretically, this important finding suggests that RS is increased (i.e., the experience of RS is potentiated) when two hands are visually-present, as though the two-hand (bilateral) system is what we are used to seeing and therefore more conducive to mediated perceptions of RS. Our setup (ART and advART) also allows much more flexibility and variety of possible therapeutic manipulations and interventions than do standard mirror boxes, as shown initially by the findings we demonstrate herein. We can therefore employ these setups to further examine extensions and a variety of features with potential positive influence on therapies which have not yet been implemented, and studies are underway towards that aim. It is known, for example, that in right handers there is a strong attentional bias to the right hand [28], [29]. Thus, even with a left-hand injury, a right visual hand might continue to naturally receive most of the participant’s attention. Its mirror reflection in an optical mirror box might not get much attention. With the optical mirror box, a participant can be asked to attend to one hand or to the other, but other than by suggestion, an attention manipulation is quite difficult to test, particularly in the case of a patient in whom attention distribution might work against positive outcomes of intended treatment (see [30]). However, with the ART setup, attention focus can easily be manipulated; for example, an image of a left hand can be placed in left space or in right space. That image can also be translated to appear as a right hand so that the patient, while feeling his left hand sensations or movement, can at the same time see what looks to be his right hand. The optimal and most efficacious treatment conditions might turn out to be idiosyncratic (as might be inferred on the basis of the extant literature using mirror boxes, given the varying proportions of participants for whom such manipulations are effective across studies). One can only know by conducting the appropriate manipulations, many of which are only possible using a video-mediated system of the type we presently have developed and are using. Our team has begun to conduct key manipulations to provide such studies, and the presented paper provides initial documentation of the comparison between setups in a group of normal controls. Notably, comfort of use was also higher in our ART setup compared to the standard OMB, suggesting the ART might be preferable in patient settings.

In summary, we find that ART has a great potential to improve therapeutic possibilities which we intend to systematically investigate on the patient groups listed in the Introduction to this paper. We have clearly shown points of difference between use of a standard optical mirror box and use of our video-mediated ART system. Further studies will examine potential benefits of this system over standard ones in the clinical setting.
